# Infectious and Noninfectious Granulomatosis in Patient with Multiple Sclerosis: Diagnostic Dilemmas and Followup

**DOI:** 10.1155/2014/876525

**Published:** 2014-02-17

**Authors:** Jelena Paovic, Predrag Paovic, Vojislav Sredovic

**Affiliations:** ^1^University Eye Clinic, Clinical Center of Serbia, Pasterova 2, 11000 Belgrade, Serbia; ^2^Primary Health Care Center, Jove Negusevica 5, 22140 Pecinci, Serbia

## Abstract

Patient was followed up over the course of 30 years. In 1978, after severe systemic infection followed by fever, pulmonary edema, and numerous neurological manifestations, patient was differentially diagnosed with apoplectic form of multiple sclerosis (MS), which was confirmed a year later via neurological and MRI findings. Approximately 20 years following the initial attack, sarcoidosis was diagnosed during the regular preoperative procedures required for cataract surgery. As consequence of lower immune system, infectious granulomatosis in form of pulmonary tuberculosis developed. Ophthalmological findings revealed bilateral retrobulbar neuritis (RBN) approximately six years after initial attack. This developed into total uveitis with retinal periphlebitis and anterior granulomatous uveitis—all of which are clinically similar in both MS and sarcoidosis.

## 1. Introduction

Sarcoidosis and multiple sclerosis (MS) belong to a group of systemic vasculitides.

Besides trauma, MS as chronic inflammation with demyelination and scarring is the most frequent cause of neurologic disability. It occurs more commonly in young middle aged females. MS, disease disseminated in time and space, is characterized by frequent recidivism. If full recovery has not been achieved, definitive neurological deficits persist. During the course of the disease, magnetic resonance imaging (MRI) reveals presence of demyelinating plaques, while liquor analysis shows oligoclonal response [1–4]. Lesions that occur in MS are characterized by perivenous cuffing and tissue infiltration by mononuclear cells (predominantly T lymphocytes and macrophages). Demyelination appears as disease progresses and as macrophages and microglial cells form the myelin debris. Due to proliferation of astrocytes, scarring occurs. Damage of myelin sheet can in most cases be connected to previous viral infection. It is believed that stress is also one of the important triggering factors [5–8].

Initial symptoms of the disease are muscle weakness in one or more limbs, blurred vision (secondary to optic neuritis), sensory disturbances, ataxia, diplopia, and so forth. However, no clinical signs or findings from diagnostic procedures are unique to MS [9, 10].

Ocular manifestations such as retrobulbar neuritis (RBN), retinal vasculitis (RV), and anterior granulomatous uveitis occur as part of MS. According to some authors, RV (periphlebitis) is a primary inflammation subsequent to vitreal inflammation and snow bank formation [3, 11].

Sarcoidosis is a multisystemic, granulomatous disease of unknown etiology. As of yet, no incriminating antigens have been proven. However, there exists an autoimmune disorder which can be manifested as noncaseating granuloma in all organs and tissue types. Due to its similarity to tuberculosis (TBC), a granulomatous infective disease characterized by caseating granulomas, many authors suggest that *Mycobacterium tuberculosis* or *Propionibacterium acnes* can influence appearance of sarcoidosis [12–14].

There are three forms of sarcoidosis: acute, subacute, and chronic. Most common systemic manifestation is pulmonary sarcoidosis, which can be manifested in four stages, that is, hilar adenopathy, pulmonary parenchyma involvement, interstitial lung changes, and chronic pulmonary fibrosis. Other forms of sarcoidosis, according to frequency of occurrence, are skin, bones and joint, mucosa, and salivary gland. Neurosarcoidosis (presence of granulomas in central nervous system (CNS)) can be manifested similarly to demyelinating diseases [15–18].

Ocular sarcoidosis occurs in 20–25% of cases who have systemic sarcoidosis. All structures of the eye can be involved, that is, cornea (dry-eye, marginal corneal infiltrates), anterior uvea (anterior granulomatous uveitis similar in characteristics to MS, viral or tuberculous anterior uveitis), and posterior segment (intermediate uveitis with both peripheral basal exudates and RV/periphlebitis) [3, 19].

There are no major differences in clinical manifestations on the eye fundus between MS and sarcoidosis in their developed states, and one can say that there is a certain degree of mimicry between the two. Intermediate uveitis and RV are ocular manifestations which occur as part of both MS and sarcoidosis but have different pathophysiological mechanisms and thus present a differential, diagnostic problem when ocular manifestations are concerned. Another differential diagnostic problem occurs due to similar appearance of inflammation on the anterior segment in a form of anterior granulomatous uveitis with mutton-fat precipitates and fibrinous exudates. There is, however, a major distinction between MS and sarcoidosis in that multifocal granulomatous posterior uveitis appears in sarcoidosis, whereas initially bilateral RBN occurs as part of MS. In both cases, late stages of disease are manifested with RV.

Ocular complications on the anterior segment in both MS and sarcoidosis are secondary glaucoma and complicated cataract. Band keratopathy is one of the complications which are characteristic for sarcoidosis alone. Possible complications on the posterior eye segment are macular edema (ME), disc atrophy, and peripheral retinal ischemia with retinal breaks and retinoschisis as a consequence. We are of the opinion that ME always occurs as complication of retinal periphlebitis, while certain authors suggest that ME occurs due to RBN [20]. In case of MS, disc atrophy can be consequence of RBN as primary ocular manifestation.

Importance of this case report lies in the fact that there are three disorders involved in case of one patient. Two of them are systemic infectious and noninfectious granulomatous diseases and one is demyelinating in nature. All three have been proven via vast clinical examinations, numerous laboratory tests, various imaging techniques (X-ray, MSCT, and MRI), histological analysis (lung biopsy), and ophthalmological examinations in one patient followedup for over 30 years (in the period from 1978 until today).

## 2. Case Report

### 2.1. 1978

Twenty-one-year old female patient was admitted to an emergency room with clinical signs of lung edema and circulatory collapse. She also had strong headache and was vomiting. Fifteen days previous to this, this female patient had suffered from strong headache, fatigue, nausea, and instability while walking. Upon admission there was no verbal communication established between her and the medical staff, and contact was highly febrile. She had problems breathing (wheezing), and was coughing up foamy, bloody, yellowish/greenish mucus. All symptoms were highly suggestive of acute pulmonary edema.

She also had tachycardia and neurological signs: right central facial palsy, left facial hyperesthesia; nystagmus; heterotopia; anisocoric pupils; asymmetric pallet and tongue deviating to the left; nasal speech; problems whilst swallowing suggestive of microlesions of the brain stem. MRI of brain and lumbar puncture revealed no pathological findings.

Laboratory analysis showed high white blood cell count, glucose level, and transaminase. Results of immunological tests for collagen and viral diseases were performed and at normal levels.

Audiometric examination pointed toward central vestibular neuritis. Ophthalmological findings were normal.

Differential diagnosis was apoplectic form of multiple sclerosis (MS).

Patient underwent treatment for pulmonary edema and tachycardia (diuretics, bronchospasmolytics, cardiotonics, corticosteroids, and antibiotics). Following treatment commencement suggestive of full recovery, there were however certain clinical signs as a repercussion of primary attack which had occurred in 1978. These repercussions were neurological in nature such as mild left facial hyperesthesia: left pallet and lip paresis; bladder incoordination; speech problems which were indicative of damage of the myelin sheath.

### 2.2. 1979

Patient returned to the emergency room with neurological symptoms similar to those she had one year earlier, but in lower intensity. She was examined by neurologist who ordered MRI and laboratory tests to exclude or confirm systemic collagen diseases or MS. MRI findings showed demyelinating changes for the first time. According to all examinations, demyelinating changes are confirmed (MS was diagnosed), while collagen diseases are excluded. She underwent systemic corticosteroid therapy which resulted in partial recovery of neurological signs.

### 2.3. 1979–1984

In this period there was no reactivation of primary disease and patient went on with normal every day activities. Two years following pregnancy, clinical signs started to appear again and subsequent ocular manifestations were evident.

### 2.4. 1984

Ocular manifestations such as painful eye movements, acute decrease of visual acuity (VA) started to appear. Complete ophthalmological examination was performed.

In April, patient's VA had slightly decreased: right eye VA from 1.0 to 0.7 and left eye VA from 1.0 to 0.9. Bilateral RBN was diagnosed. Uveitis was not found. Patient received pulse doses of corticosteroid agent (repeated doses of metilprednisolone, 1000 mg, intravenous).

In October second lumbar puncture was performed due to confirming diagnosis of MS. Intrathecal synthesis antibodies were not confirmed.

Six months after diagnosis of RBN, first bilateral inflammatory changes of uveal tissue in form of total uveitis were diagnosed—proteins and cellular Tyndall in anterior chamber and vitreous, inflamed retinal blood vessels (mostly veins), papillitis, and macular (ME) and retinal edema (RE). According to all these ocular manifestations, disease was diagnosed as neurouveitis. Treatment consisted of systemic and local application of corticosteroid agents.

### 2.5. 1989

After 5 years of remission, patient was admitted to the neurology ward because of transitory speech problems, weakness of right hand, and urine incontinency. MRI was performed once again and showed diffused, high intensity, bilateral, periventricular, subcortical, and deep changes in white matter of CNS. NMR finding in conjunction with neurological signs indicated that there was MS present (approximately 11 years following an initial diagnosis when it was assumed that the patient had apoplectic form of MS).

Ophthalmological manifestations were persistent, recurrent, bilateral uveitis, ME (as consequence of RV), and disc atrophy (as consequence of RBN) which lead to decrease of visual acuity. Once again, patient received repeated pulse doses of corticosteroids.

### 2.6. 1989–2003

During this period, patient did not consult neurologist. She had had, however, regular consultations with an ophthalmologist regarding uveitis.

### 2.7. 2003

It was noted that besides other symptoms patient still had some speech impairment; paresthesias; impaired muscle coordination; from time to time loss of sensation; impaired vision; urinary incontinence and additional tests were performed in order to once again check the etiology of the disease. Immunological tests for collagen and viral diseases were repeated and only antinuclear antibody-HEp-2 test was positive.

Progressions of uveitis lead to development of bilateral complicated cataract.

### 2.8. 2004

While undergoing tests required for cataract surgery it was suspected that the patient might be suffering from sarcoidosis. Among other tests, chest X-ray revealed fibrinous, bilateral, lung changes and subsequently granulomatosis and chronic lung sarcoidosis of 3rd degree. Angiotensin converting enzyme (ACE) was noted to be 68 U/L (normal range 8–52 U/L).

Biopsy finding of bronchial mucosa showed pathophysiological changes in lamina propria (basement membrane) in form of small granulomas consisting of few changed histiocytes, lymphocyte, individual Langerhans type giant cells, plasma cells eosinophils, and granulocytes. Necrosis has not been noted. Mild interstitial fibrosis was present in the pulmonary/lung parenchyma typical for noncaseating granulomatosis (sarcoidosis). Tuberculosis (TB) was excluded.

Due to presence and confirmation of pulmonary sarcoidosis, CNS MRI was repeated in order to investigate neurosarcoidosis. MRI revealed diffused, multifocal lesions which affected both cerebral hemispheres and cerebellum (its' white matter was depicted). These changes were assumed to be demyelinating in nature as there was presence of consecutive, second degree, global, white matter atrophy. Active lesions or manifestations of neurosarcoidosis were not found. Seeing that liquor test was negative and that there was a benign clinical picture but that there was evidence of demyelination, secondary, CNS, demyelinating vasculitis was suspected.

As part of preoperative management, patient received local and systemic corticosteroid drugs after which bilateral phacoemulsification cataract surgery was performed.

### 2.9. 2004–2009

During this period patient consulted pulmonologist regarding lung sarcoidosis, and ophthalmologist regarding ocular manifestations and received systemic as well as local treatments as per protocol (corticosteroid and/cytostatic drugs). No neurological consultations were performed during this period.

**Figure 1:**
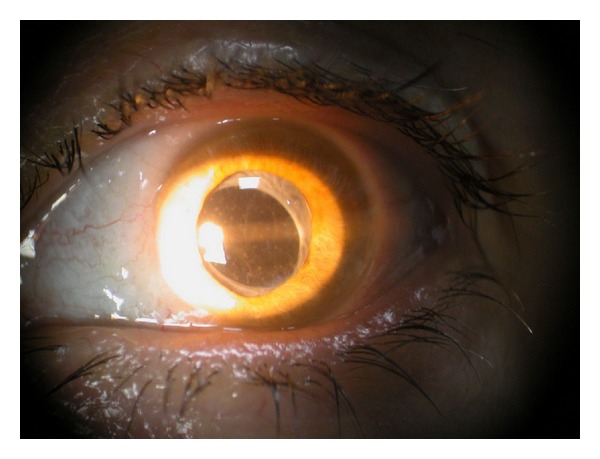
Pseudophakia, secondary cataract, and anterior granulomatous uveitis in MS (right eye).

**Figure 2:**
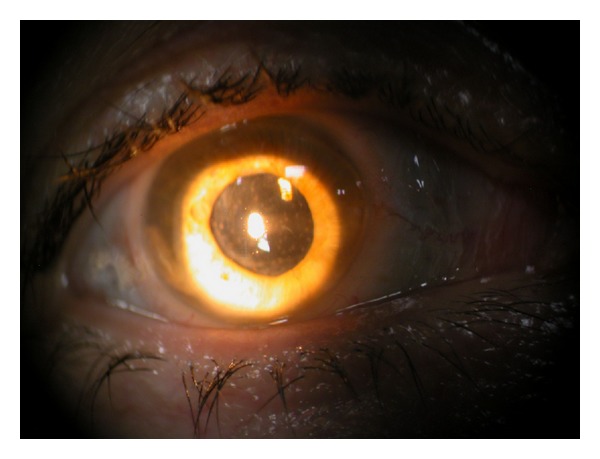
Pseudophakia, secondary cataract, and anterior granulomatous uveitis in MS (left eye).

**Figure 3:**
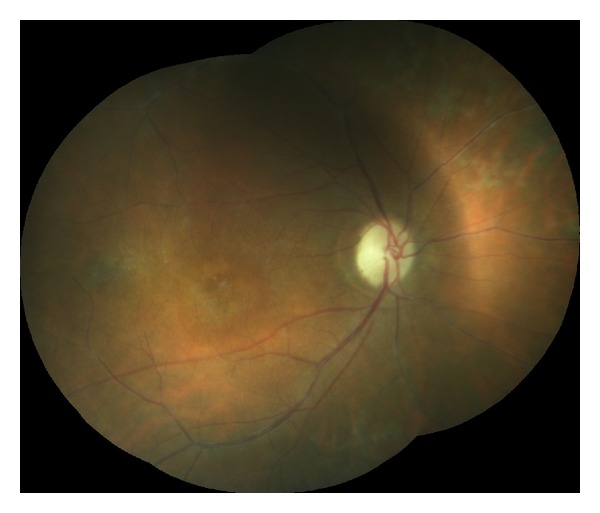
Retinal vasculitis, disc atrophy, and macular edema in patient with MS (right eye).

**Figure 4:**
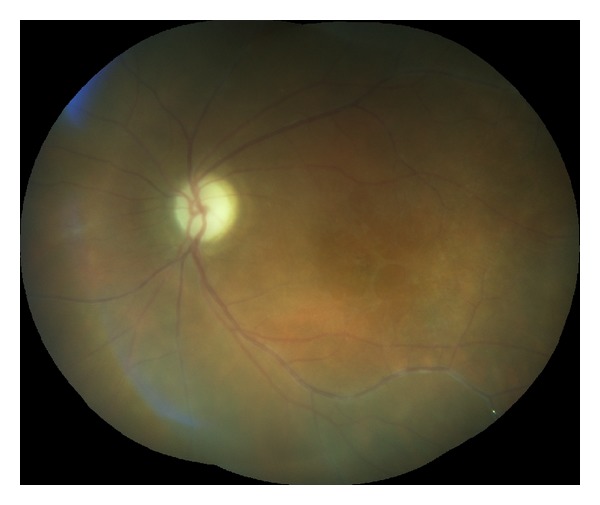
Retinal vasculitis, disc atrophy, and macular edema in patient with MS (left eye).

**Figure 5:**
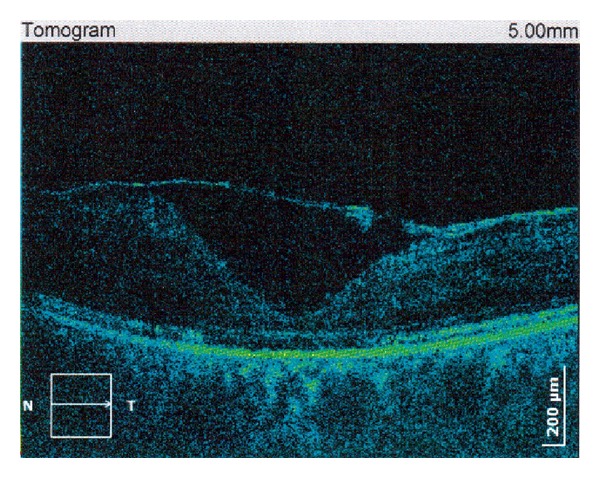
Macular edema and epiretinal membrane as complication of periphlebitis in patient with MS (right eye).

**Figure 6:**
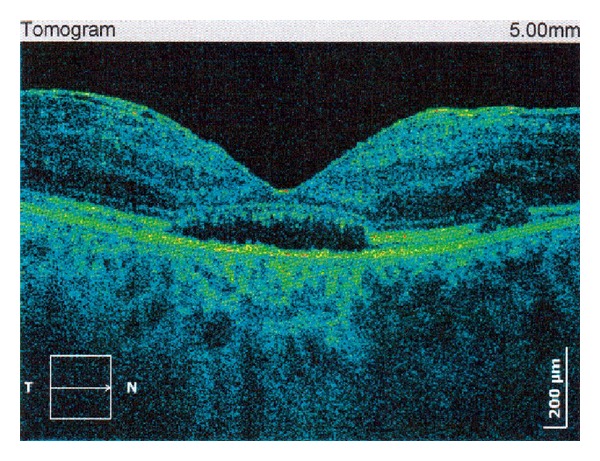
Macular edema and epiretinal membrane as complication of periphlebitis in patient with MS (left eye).

**Figure 7:**
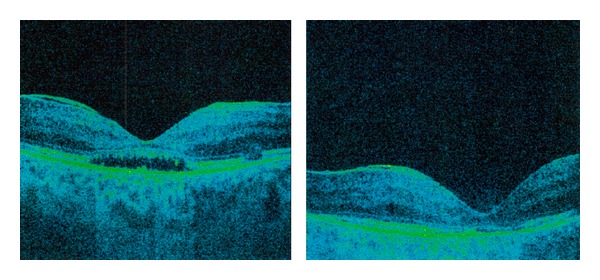
Macular edema, subretinal fluid, and epiretinal membrane treated with sub-Tenon's triamcinolone acetonide injections in patient with retinal vasculitis in MS (followup).

Over the course of this period, patient experienced recurrent bilateral uveitis and thus as a consequence had secondary cataract and posterior capsulotomy was performed (Figures 1 and 2). There were repeated cycles of RV as well (Figures 3 and 4). Periphlebitis occurred more frequently than periarteritis. ME and epiretinal membranes appeared as complications of RV (Figures 5 and 6). Repeated sub-Tenon's injections of triamcinolone acetonide (40 mg every 3-4 weeks) were used in treatment of these complications (Figure 7).

### 2.10. 2009

In order to confirm sarcoidosis extensive testing was performed among which were chest X-ray and MSCT scan. MSCT imaging showed intense condensation of pulmonary tissue of the right top lobule in form of mixed reticular/nodular type with pronounced fibrosis of rarer, nodular fields predominantly peripherally located. Peribronchial fibrosis, with hilo lateral as well as hilo apical, multidirectional, distribution was noted. Partially wet necrotic lymphoid nodules were present in both the subcarinal right side of mediastinum and along the spine. Two laboratory readings showed increased levels of ACE in serum (86 U/L, 65 U/L, resp.).

### 2.11. 2010

During the course of the year, patient exhibited neurological problems, while towards the end of the year she noted blood in sputum which led to further testing for sarcoidosis. ACE serum level was 68 U/L. As there were signs indicative of neurosarcoidosis, MRI was repeated and revealed presence of consequential, supratentorial lesions of open etiology, which could differentially point towards periventricular leukoencephalopathy. Due to this, neurosarcoidosis was not confirmed, but it rather stayed as differential diagnosis.

### 2.12. 2011

Due to additional clinical signs indicative of pulmonary tuberculosis (TBC), additional tests were performed. Microscopic sputum examination confirmed presence of acid-fast bacilli. Laboratory tests showed high blood sedimentation rate, CRP, and leukocyte count. Based on previous results and current examinations, patient was diagnosed with pulmonary TBC and underwent anti-TBC treatment (as per protocol). Initial treatment during the period of 2 months consisted of isoniazid, rifampicin, pyrazinamide, and ethambutol. For the next four months isoniazid and rifampicin were applied. Patients liver and kidney functions were monitored regularly during this period. Within three months, remission of the disease was noted.

From onset of the disease until today, ophthalmological examinations revealed no TBC ocular manifestations present.

### 2.13. 2013

In August of this year, due to exacerbation of old and appearance of new neurological symptoms (such as uncoordinated movements), patient was admitted to neurology ward and subjected to further examinations. Latest MRI images showed no new changes as compared to previous findings from 2010. Patient was released from hospital with intent to return in 3-4 months in order to undergo further diagnostic testing. During this period she was advised to monitor her physical condition and take therapy required for circulatory disease (Ginkgo biloba extract, 40 mg every 8 hours) and spasmolytic drugs (tolterodine tartrate, 2 mg every 12 hours).

## 3. Discussion

This case report is indicative of a complex diagnostic problem which can present itself in the field of medicine. Here we have a case where the onset of the disease was acute in nature and in a manner that is typical for a systemic infection that led to substantial damage of various CNS structures and a suspicion of apoplectic form of MS.

Even though the first (initial) diagnostic procedures such as MRI did not fully prove demyelinating changes, neurologically persistent changes and later confirmation of demyelinating plaque all point towards the fact that the patient had MS from the very beginning. Demyelinating optic neuritis is often a herald of MS but is only one of many possible neuroophthalmologic abnormalities which may occur. Demyelinating white matter lesions that can be present in the brain MRI at the time of presentation of optic neuritis are the strongest predictor for development of clinically definite MS. In most cases with optic neuritis there are white matter lesions that are present and are consistent with this diagnosis. In favor of a MS is also the fact that there was RBN present at the time and this is one of the most common and amongst the first ocular manifestation of MS. It is possible that one of the pathogens, most probably of viral etiology, caused damage to the myelin sheath and led to development of the demyelination.

Few years later pulmonary sarcoidosis which has been proven via X-ray, MSCT and has histological proof to back it up cannot be connected to MS, but it can, however, be connected to previous systemic infection. So, this poses a differentially diagnostic problem. Namely, bearing in mind that sarcoidosis and MS have similar MRI and neurological manifestations, and that there have been certain CNS symptoms noted, was there actually neurosarcoidosis present as part of systemic sarcoidosis at the time?

During the course of the disease, pulmonary TB developed and was proven via numerous laboratory tests and microscopic analysis, only to be resolved six months following its initial appearance and prescribed treatment. Such outcome could be attributed to the fact that there was a decline in patients' immune response due to a lengthy immunosuppressive therapy consisting of various corticosteroids and cytostatic drugs. Besides diagnostic and differentially diagnostic issues which have arisen in this case, a particular problem lies in application of treatment between the two diseases. In particular we have noninfectious granulomatosis, where primary therapy consists of corticosteroids, and infectious granulomatosis, where primary therapy consists of agents belonging to a completely opposite group of medicaments (noncorticosteroid therapy). Problem lies in the fact that corticosteroids are contraindicative in treatment of TBC. Thus it is particularly interesting that there is incidence of both infective and noninfective granulomatosis present at the same time in the same individual.

Ophthalmic assessment was very interesting in itself and suggested that the disease began as bilateral RBN followed by optic disc atrophy and a later development of neurouveitis, all typical for infectious diseases and of demyelinating processes. Later on, the main ocular, clinical manifestation was in form of bilateral RV (predominantly periphlebitis) as well as anterior granulomatous uveitis (which can develop as part of both MS and sarcoidosis).

Clinical signs are suggestive of the fact that there was an inflammation caused by systemic infection or autoimmune process in the eye where the pathogenic reagent was a trigger factor, whereas inflammation of the optic disc and retinal blood vessels points towards a demyelinating process (a fact which was proven correct via an MRI imaging performed in 1989, which was nearly 11 years following the initial attack).

Recurrent and progressive course of disease has resulted in severe complications some of which are in form of a complicated cataract (which was operated on) and ME, all of which seem to respond well to local (sub-Tenon's injections of triamcinolone acetonide) and systemic (mainly corticosteroid) therapy. Patient's history as well as clinical signs of the disease represents a major diagnostic and therapeutic challenge requiring multidisciplinary approach and frequent monitoring.

Until today, in this particular case one cannot say with 100% certainty that ocular and other manifestations are typical signs of MS and sarcoidosis but are rather a possible consequence of a systemic infection which had started in 1978 and developed into these, previously mentioned diseases.
